# Meeting of the American Medical Association

**Published:** 1868-04

**Authors:** 


### Editorial Department.

### Meeting of the American Medical Association.

It will be seen by the notice of the Secretary that the American Medical Association holds its annual meeting, this year, in Washington, and it was last year voted to make Washington its place of meeting every second year. Since the outbreak of the Rebellion the profession in the South have been but feebly and at times not at all represented. Washington is a central location, and from its position invites a full and universal representation. The South is now professionally "re-constructed," and we can greet our Southern friends with a hearty cordiality. Science knows no party, sect, creed, or nationality, and the real bond of brotherhood in the American medical profession has never been broken or weakened by our political and sectional strifes; our avenues of communication have been blockaded, but the chain of sympathy and good-fellowship has never been separated. Whoever has faithfully labored for the truth, made discovery in science, or added to the value of our art, is always and everywhere recognized with pride and pleasure. If the physicians of the South do not hold to our political views they will not be "impeached;" they may "worship God according to the dictates of their own consciences," and while they practice rational medicine, and seek to advance it, "they can have our hand," as physicians.

But this is no part of what we designed to say when this meeting was introduced to the attention of our readers. We only propose to say a word of what this Association has already accomplished, what still remains to be done, the claims it has to support, and how these obligations should be discharged. To show what this Association has accomplished and what remains to be done, it is only necessary to remember the objects had in view at the time of its organization, now, some twenty-two years since, and observe how much remains to be done—the progress which has really been made. The American Medical Association was designed to be instituted upon a plan and conducted in such manner "as to give frequent, united and emphatic expression to the views and aims of the medical profession in this country, to at all times have a beneficial influence and supply more efficient means than had hitherto been available here, for cultivating and advancing medical knowledge, for elevating the standard of medical education, for promoting the usefulness, honor and interests of the medical profession; for enlightening and directing public opinion in regard to the duties, responsibilities and requirements of medical men, for exciting and encouraging emulation and concert of action in the profession, and for facilitating and fostering friendly intercourse between those who are engaged in it." There are few physicians who will not cheerfully and gladly acknowledge that these objects have already been answered in greater or less degree. Its code of medical ethics, its annual volume of transactions, containing many of the most valuable contributions to medical literature, made in this, or any other country, its efforts and influence in raising the standard of medical education, its opportunities of friendly intercourse, and the influence it has always had in "directing and enlightening" public opinion in regard to the duties, responsibilities and requirements of medical men, all show how wisely it was planned, and how successfully it has accomplished the objects held prominently in view.

It will not be thought that any of these objects have been fully and satisfactorily accomplished, so that nothing more remains to be done; its work is a reformation of progress which shall be eternal, slow but sure. Impatient and dissatisfied grumblers charge upon it incapacity for good to the general profession, and useful only to a few, failure in accomplishing its proposed objects, and of no importance in its general influence with either the profession or public. This is all very well, it only furnishes additional and positive proofs of its prosperity, usefulness and success. We most sincerely hope that the profession of the country will be fully represented, and should be proud if the transactions of the Association should be enriched by contributions from some of the earnest, thinking, practical men of our own city and of Western New York.

AMERICAN MEDICAL ASSOCIATION.

Office of Permanent Secretary,
Wm. B. Atkinson, M. D.,
S. W. cor. Broad & Pine sts.,
Philadelphia, Pa.

The Nineteenth Annual Meeting of the American Medical Association will be held in Washington, on Tuesday, May 5th, 1868, at 11 o'clock A. M.

The following Committees are expected to report:

On Ophthalmology, Dr. Joseph S. Hildreth, Illinois, Chairman.

On Cultivation of the Cinchona Tree, Dr. J. M. Toner, D. C., Chairman.

On Surgical Diseases of Women, Dr. Theophilus Parvin, Indiana, Chairman.

On Rank of Medical Men in the Navy, Dr. N. S. Davis, Illinois, Chairman.

On Insanity, Dr. C. A. Lee, New York, Chairman.

On American Medical Necrology, Dr. C. C. Cox, Maryland, Chairman.

On Leakage of Gas Pipes, Dr. J. C. Draper, New York, Chairman.

On Medical Ethics, ———, Chairman.

On Plan of Organization, Dr. C. C. Cox, Maryland, Chairman.

On Provision for the Insane, Dr. C. A. Lee, New York, Chairman.

On the Climatology and Epidemics of Maine, Dr. J. C. Weston; of New Hampshire, Dr. P. A. Stackpole; of Vermont, Dr. Henry Janes; of Massachusetts, Dr. Alfred C. Garrett; of Rhode Island, Dr. C. W. Parsons; of Connecticut, Dr. E. K. Hunt; of New York, Dr. W. F. Thoms; of New Jersey, Dr. Ezra M. Hunt; of Pennsylvania, Dr. D. F. Condie; of Maryland, Dr. O. S. Mahon; of Georgia, Dr. Juriah Harriss; of Missouri, Dr Geo. Engelman; of Alabama, Dr. R. Miller; of Texas, Dr. T. J. Heard; of Illinois, Dr. R. C. Hamil; of Indiana, Dr. J. F. Hibberd; of District of Columbia, Dr. T. Antisell; of Iowa, Dr. J. W. H. Baker; of Michigan, Dr. Abm. Sager; of Ohio, Dr. J. W. Russell; of California, Dr. F. W. Hatch; of Tennessee, Dr. Joseph Jones; of West Virginia, Dr. E. A. Hildreth; of Minnesota, Dr. Samuel Willey.

On Clinical Thermometry in Diphtheria, Dr. Jos. G. Richardson, New York, Chairman.

On the Treatment of Disease by Atomized Substances, Dr. A. G. Field, Iowa, Chairman.

On the Ligation of Arteries, Dr. Benj. Howard, New York, Chairman.

On the Treatment of Club-Foot without Tenotomy, Dr. L. A. Sayer, New York, Chairman.

On the Radical Cure of Hernia, Dr. G. C. Blackman, Ohio, Chairman.

On Operations for Hare-Lip, Dr. Hammer, Missouri, Chairman.

On Errors of Diagnosis in Abdominal Tumors, Dr. G. C. E. Weber, Ohio, Chairman.

On Prize Essays, Dr. Charles Woodward, Ohio, Chairman.

On Medical Education, Dr. A. B. Palmer, Michigan, Chairman.

On Medical Literature, Dr. George Mendenhall, Ohio, Chairman.

Secretaries of all medical organizations are requested to forward lists of their delegates as soon as elected, to the Permanent Secretary.

W. B. ATKINSON.

